# Multilocus Molecular Characterization of a 16SrII-D Phytoplasma Infecting Black Carrot in Türkiye

**DOI:** 10.3390/pathogens15070712

**Published:** 2026-07-07

**Authors:** Hakan Çarpar, Ömer Faruk Coşkun

**Affiliations:** 1 Department of Plant Protection, Faculty of Agriculture, Hatay Mustafa Kemal University, Hatay 31060, Türkiye; hcarpar@mku.edu.tr; 2 Department of Horticulture, Faculty of Agriculture, Hatay Mustafa Kemal University, Hatay 31060, Türkiye

**Keywords:** black carrot, phytoplasma, 16SrII-D, *secA*, *tuf*, *imp*, SAP11, phylogenetic analysis

## Abstract

During the 2024 growing season, black carrot (*Daucus carota* subsp. *sativus*) plants showing symptoms consistent with phytoplasma infection, including leaf chlorosis, reduced leaf size, witches’ broom, excessive fibrous root formation, multiple lateral taproots, and floral phyllody, were observed in production fields in Hatay Province, Türkiye. To identify the associated phytoplasma, 23 symptomatic plants and two asymptomatic control plants were analysed using PCR-based molecular detection, sequencing, BLASTn comparison, and phylogenetic analyses of the 16S rRNA, *secA*, *tuf*, and *imp* gene regions. The SAP11 gene was also screened as an additional virulence-associated molecular marker, but no functional characterization was performed. All symptomatic samples yielded amplicons of the expected sizes for the targeted loci, whereas no amplification was obtained from asymptomatic controls. Sequence comparisons revealed >99% nucleotide identity with members of the peanut witches’ broom group, and multilocus phylogenetic analyses consistently placed the black carrot phytoplasma isolate within the 16SrII-D subgroup. To our knowledge, this study provides the first documented evidence of a 16SrII-D phytoplasma associated with black carrot in Türkiye. The finding is relevant for plant pathology and plant protection because it indicates the occurrence of a phytoplasma lineage with potential epidemiological importance in an economically important vegetable production area. These results provide a basis for future studies on disease distribution, insect vectors, alternative host plants, epidemiology, and management strategies in black carrot production systems.

## 1. Introduction

Carrot (*Daucus carota* L.) is an economically important vegetable crop cultivated worldwide for both fresh consumption and industrial processing. Black carrot (*Daucus carota* subsp. *sativus*) has particular industrial and economic relevance because its storage roots are rich in anthocyanins, which are widely used as natural colorants in food and beverage industries. Global carrot and turnip production exceeds 41 million tons annually, and Türkiye is among the leading producers, with Hatay representing one of the major black carrot production areas [1,2]. However, productivity and quality are constrained by numerous biotic and abiotic factors, among which phytoplasma diseases have emerged as important threats to sustainable vegetable production. Diseases that impair root development, marketable root quality, or pigment accumulation may reduce both fresh-market value and industrial processing quality. Phytoplasma-associated symptoms such as excessive fibrous rooting, lateral taproot formation, chlorosis, and phyllody may directly compromise root morphology, harvestable yield, and the quality traits that determine the commercial value of black carrot.

Phytoplasmas are wall-less, phloem-limited plant pathogens belonging to the class Mollicutes and are associated with substantial economic losses in a wide range of crops worldwide [3,4]. Infected plants typically exhibit symptoms such as chlorosis, stunting, witches’ broom, virescence, phyllody, and root abnormalities [3]. These pathogens are mainly transmitted by phloem-feeding insects, particularly leafhoppers and planthoppers, which facilitate disease spread within and between production areas [5,6]. Since phytoplasmas cannot be cultured in vitro, their detection, classification, and epidemiological investigation rely largely on molecular approaches [7]. The 16Sr group/subgroup classification system, based mainly on restriction fragment length polymorphism or virtual RFLP analysis of 16S rRNA gene sequences, remains widely used for routine identification and epidemiological studies [8,9,10]. However, the revised ‘*Candidatus* Phytoplasma’ species description guidelines increased the 16S rRNA gene sequence identity threshold for species demarcation from 97.5% to 98.65%, and additional molecular, genomic, or biological evidence is required when closely related taxa exceed this value [7]. Because the 16S rRNA gene is relatively conserved, additional loci such as *secA*, *tuf*, and *imp* are useful for improving phylogenetic resolution and supporting subgroup-level discrimination [11,12]. Compared with the highly conserved 16S rRNA gene, *secA* and *tuf* provide greater discriminatory power for resolving relationships among closely related phytoplasma lineages, while *imp*, which encodes an immunodominant membrane protein, may offer additional strain-level and subgroup-level resolution. These loci have been used in previous phytoplasma taxonomic and phylogenetic studies to complement 16S rRNA-based classification and to strengthen subgroup assignment, particularly when closely related 16Sr lineages are involved.

Phytoplasma diseases of carrot have been reported in many regions of the world and have been associated with several phytoplasma groups, including 16SrI, 16SrV, 16SrVI, and 16SrXII [3,13]. In Türkiye, stolbur phytoplasma (16SrXII-A) has previously been reported in carrot fields in Hatay province, while 16SrVI phytoplasmas were detected in carrot seeds and alternative hosts in Ankara and Konya provinces [14,15]. In contrast, reports of peanut witches’ broom group phytoplasmas (16SrII) infecting carrot remain relatively limited and are mainly confined to the Middle East and Asia. Previous studies in Iran, India, and Indonesia demonstrated the occurrence of 16SrII phytoplasmas associated with witches’ broom, phyllody, virescence, and yellowing symptoms in carrot [16,17,18]. These findings indicate that carrot can serve as a suitable host for different members of the 16SrII lineage.

Beyond taxonomic identification, phytoplasma pathogenicity may involve secreted effector proteins that interfere with host developmental and defense pathways. Among these effectors, SAP11 is one of the best-characterized virulence factors and has been associated with shoot proliferation, altered leaf development, and suppression of jasmonate-mediated defense responses through interactions with plant TCP transcription factors [19,20]. In the present study, SAP11 was included only as an additional virulence-associated screening marker, not as a target for functional characterization. Furthermore, the 16SrII group is among the important phytoplasma lineages reported in the Middle East and has been detected in several vegetable, legume, and weed hosts [21,22]. The broad host range and epidemiological importance of the 16SrII-D subgroup highlight the need for continuous monitoring of this phytoplasma lineage in regional production systems.

Although phytoplasmas have been extensively investigated in several crops in Türkiye, no confirmed report has documented the occurrence of a peanut witches’ broom phytoplasma belonging to subgroup 16SrII-D in black carrot. To our knowledge, this is the first documented report of a 16SrII-D phytoplasma associated with black carrot in Türkiye. This finding is important for regional plant health monitoring because it documents the presence of an epidemiologically relevant phytoplasma subgroup in one of the country’s major black carrot production areas and establishes a foundation for future investigations on disease distribution, insect vectors, alternative host plants, and disease management. Therefore, the objective of the present study was to identify and molecularly characterize the phytoplasma associated with phytoplasma-like symptoms observed in black carrot fields in Hatay Province during the 2024 growing season. To strengthen subgroup-level identification, analyses were based on the 16S rRNA, *secA*, *tuf*, and *imp* gene regions, while the virulence-associated SAP11 gene was additionally screened. PCR amplification, sequencing, BLASTn analysis, and phylogenetic approaches were employed to determine the taxonomic position of the detected phytoplasma and to provide robust multilocus evidence supporting its assignment to subgroup 16SrII-D.

## 2. Materials and Methods

### 2.1. Plant Material and Sampling

**Figure 1:**
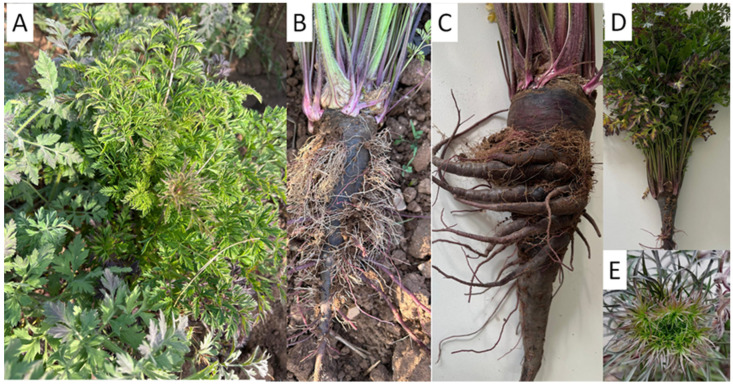
Symptoms observed in infected plants: (**A**,**B**,**D**) leaf chlorosis accompanied by a marked reduction in leaf size and shoot proliferation resulting in a witches’ broom-like appearance; (**B**,**C**) extensive fibrous root development on the taproot and formation of multiple lateral taproots; (**E**) phyllody observed in floral organs.

This study was conducted during the 2024 growing season in black carrot production fields in Hatay Province, Türkiye (Kırıkhan; 36°28′36.1″ N 36°22′14.6″ E). Surveys were conducted in five commercial fields within Kırıkhan district; the coordinates given correspond to the central point of the surveyed area. Fields were surveyed for plants showing phyllody, witches’ broom, chlorosis, and abnormal root development. Symptoms recorded in the field included leaf chlorosis and reduced leaf size, shoot proliferation, excessive fibrous root formation on the taproot, development of multiple lateral taproots, and floral phyllody (Figure 1). For molecular analyses, samples were collected from 23 symptomatic plants. Additionally, two asymptomatic plants from the same fields were sampled as negative controls. Young leaves showing clear symptom expression were preferentially excised using sterile scissors or scalpels, placed in sterile sampling bags, transported to the laboratory under cold-chain conditions, and stored at −20 °C until DNA extraction. Sampling was based on purposive selection of plants exhibiting clear and representative phytoplasma-like symptoms, including leaf chlorosis, reduced leaf size, witches’ broom, excessive fibrous root formation, multiple lateral taproots, and phyllody. To verify symptom-associated pathogen detection, two apparently healthy plants from the same production fields were also sampled as asymptomatic negative controls. Plant materials used in this study were collected directly from commercial black carrot production fields during routine disease surveys conducted with the permission of field owners. No protected or endangered plant species were involved, and sampling complied with local agricultural regulations.

### 2.2. Total DNA Extraction

Total DNA was isolated from leaf samples using a CTAB-based procedure adapted from Doyle and Doyle [23]. Approximately 0.5 g of fresh tissue per plant was pulverized in liquid nitrogen and immediately combined with prewarmed CTAB extraction buffer. Following extraction, DNA was recovered by isopropanol precipitation, rinsed with 70% ethanol, and the pellet was allowed to dry at room temperature before being dissolved in 100 µL of nuclease-free water or TE buffer. Isolated DNA quality was verified by electrophoresis on 1% agarose gels, while DNA yield and purity were quantified using a NanoDrop spectrophotometer. The isolated DNA samples were subsequently used as PCR templates.

### 2.3. PCR Detection of Phytoplasma and Amplification of Target Gene Regions

Phytoplasma detection and molecular characterization were performed by PCR targeting the 16S rRNA gene and additional phytoplasma genetic markers, including *secA*, *tuf*, *imp*, and SAP11. The SAP11 gene was included as an additional virulence-associated marker for PCR-based screening. Detection of phytoplasma 16S rRNA followed a two-step approach consisting of a first-round PCR using P1/P7 primers, followed by nested PCR with R16F2n/R16R2 to increase detection sensitivity and obtain the F2n/R2 fragment commonly used for 16Sr group and subgroup identification [24,25]. The *secA*, *tuf*, *imp*, and SAP11 gene regions were amplified directly from total DNA extracts. Detailed information regarding primer pairs, expected amplicon sizes, and references is presented in Table 1. PCR reactions were performed in a final volume of 25 µL containing 1× PCR buffer, 2.5 mM MgCl_2_, 0.2 mM of each dNTP, 0.2 µM of each primer, 1.0 U Taq DNA polymerase (Thermo Fisher Scientific, Waltham, MA, USA), and approximately 50–100 ng of template DNA. All primer pairs were amplified using an annealing temperature of 55 °C. The PCR program consisted of an initial denaturation at 94–95 °C for 2–5 min, followed by 35 cycles of denaturation at 94–95 °C for 30 s, annealing at 55 °C for 30 s, and extension at 72 °C for 45–60 s, with a final extension step at 72 °C for 5–10 min. Each PCR run included DNA extracted from asymptomatic plants and a no-template control containing nuclease-free water. A positive control was not available for this survey. Amplification products were separated on 1.5% agarose gels, stained with SYBR Safe, visualized under UV illumination, and sized using a GeneRuler 100 bp DNA Ladder (Thermo Fisher Scientific, Waltham, MA, USA).

### 2.4. Sequencing, Sequence Editing, and BLAST Analysis

Amplicons of the expected size obtained from all 23 symptomatic samples were excised from agarose gels, purified using a gel extraction procedure, and subjected to bidirectional Sanger sequencing for all five targeted loci (16S rRNA, *secA*, *tuf*, *imp*, and SAP11) using the corresponding forward and reverse primers by BM Labosis Biotechnology (Ankara, Türkiye). Chromatograms were inspected and edited using MEGA X (version 10.2.6), and low-quality terminal regions were trimmed prior to sequence assembly. Forward and reverse reads were assembled to generate consensus sequences for each locus from each symptomatic sample. The resulting consensus sequences were compared among samples, and no sequence polymorphism was detected for any of the analysed loci. Consequently, one representative consensus sequence per locus was selected for GenBank submission and subsequent phylogenetic analyses. Representative sequences were compared with those available in the NCBI GenBank database using the NCBI BLASTn web server (https://blast.ncbi.nlm.nih.gov/Blast.cgi) (accessed on 5 May 2026), and nucleotide identities were determined based on the closest-matching reference sequences. The 16Sr group/subgroup affiliation was inferred by comparison of the 16S rRNA and additional marker gene sequences with appropriate phytoplasma reference sequences.

### 2.5. Phylogenetic Analyses

Representative consensus sequences of the 16S rRNA, *secA*, *tuf*, and *imp* gene regions generated in this study were aligned with representative phytoplasma reference sequences retrieved from the NCBI GenBank database, and separate phylogenetic trees were constructed for each locus. Multiple sequence alignments were performed using ClustalW, and Maximum Likelihood analyses were conducted in MEGA X (version 10.2.6). Branch support was assessed using 1000 bootstrap replicates. Phylogenetic trees were rooted with *Acholeplasma laidlawii* (Acc. No. FJ226570) for the 16S rRNA dataset, Bacillus subtilis (Acc. No. D10279) for the *secA* dataset, *Ureaplasma parvum* (Acc. No. AF270770) for the *tuf* dataset, and *Phytoplasma* sp. (Acc. No. MG435348) for the *imp* dataset. As no sequence polymorphism was detected among the 23 symptomatic samples, one representative consensus sequence per locus was selected for phylogenetic reconstruction. The multilocus phylogenetic analyses were performed to independently verify the subgroup assignment inferred from the 16S rRNA gene and to evaluate whether the same taxonomic placement was consistently supported across multiple genetic loci. They were not intended to assess population-level genetic diversity, recombination, or detailed evolutionary relationships among isolates.

### 2.6. Data Evaluation

Molecular diagnostic outputs, including PCR amplification, bidirectional sequencing, BLASTn results, and phylogenetic clustering, were interpreted together with field symptom observations. Concordance among loci was evaluated by assessing whether symptomatic samples yielded consistent amplification across the targeted gene regions and whether their sequences showed the highest similarity to phytoplasma reference sequences available in the NCBI GenBank database. The sequences generated in this study were deposited in GenBank under accession numbers PX482184 (16S rRNA), PZ104026 (*imp*), PZ104028 (*secA*), PZ104029 (*tuf*), and PZ104027 (SAP11). Assignment to subgroup 16SrII-D was based on 16S rRNA gene analysis and independently supported by concordant multilocus sequence comparisons and phylogenetic analyses.

The absence of a positive PCR control was recognized as a limitation of the diagnostic workflow. To minimize diagnostic uncertainty, each PCR assay included DNA extracted from asymptomatic plants and a no-template control. Furthermore, diagnostic confidence was strengthened by the consistent amplification of five independent genetic markers in all symptomatic samples, the absence of amplification in negative controls, bidirectional Sanger sequencing, high BLASTn sequence identity with reference phytoplasmas, and concordant phylogenetic placement within the 16SrII-D subgroup. Future studies will include a validated reference isolate or reference DNA as a positive control to further improve assay validation and diagnostic reliability.

## 3. Results

### 3.1. Description of Symptoms

During field surveys conducted in 2024 in black carrot (*Daucus carota* subsp. *sativus*) production fields in Hatay province, plants showing symptoms consistent with phytoplasma infection were observed. Symptomatic plants commonly exhibited leaf chlorosis accompanied by a clear reduction in leaf size, together with shoot proliferation giving a witches’ broom-like appearance. In the root system, abundant fibrous root development on the taproot and the formation of multiple lateral taproots were recorded. Floral organs also showed phyllody (Figure 1). These symptoms were documented at varying intensities in all 23 symptomatic plants sampled in this study. Since the present work was designed for molecular detection and multilocus characterization rather than disease-incidence assessment, the precise field-level infestation rate was not quantified during the survey.

### 3.2. PCR Detection of Phytoplasma

All symptomatic samples produced the expected first-round P1/P7 amplicon (1.8 kb) and yielded the expected nested F2n/R2 product (1.25 kb). PCR assays targeting the *secA*, *tuf*, and *imp* gene regions generated products of the expected lengths in all 23 symptomatic samples. The SAP11 gene was also successfully amplified in symptomatic samples, confirming its presence. In contrast, no amplification was obtained from DNA extracted from the two asymptomatic plants used as negative controls, nor from the no-template control (NTC).

### 3.3. Sequence Analysis and BLASTn Results

PCR products obtained from all 23 symptomatic samples were sequenced bidirectionally for all five targeted loci (16S rRNA, *secA*, *tuf*, *imp*, and SAP11), and consensus sequences were generated for each locus. Sequence comparisons among the symptomatic samples were performed to identify possible nucleotide variation, including single-nucleotide polymorphisms (SNPs) and other sequence polymorphisms, within the amplified regions. No sequence polymorphism was detected for any of the analysed loci, indicating complete sequence identity across the targeted marker regions. BLASTn analysis showed that the 16S rRNA, *secA*, *tuf*, *imp*, and SAP11 sequences shared nucleotide identities ranging from >99% to 100% with phytoplasma reference sequences belonging to the peanut witches’ broom group (16SrII) available in the NCBI GenBank database. For all analysed loci, the closest matches corresponded to reference isolates assigned to the 16SrII-D subgroup. The SAP11 sequences also showed high nucleotide identity with SAP11 sequences of previously reported 16SrII phytoplasmas, and no sequence variation was detected among the symptomatic samples. Because identical sequences were obtained from all symptomatic plants for each analysed locus, one representative consensus sequence per locus was selected for GenBank submission, BLASTn analysis, and phylogenetic reconstruction. The representative sequences were deposited in GenBank under accession numbers PX482184 (16S rRNA), PZ104026 (*imp*), PZ104028 (*secA*), PZ104029 (*tuf*), and PZ104027 (SAP11).

### 3.4. Phylogenetic Analysis Based on the 16S rRNA Gene

**Figure 2:**
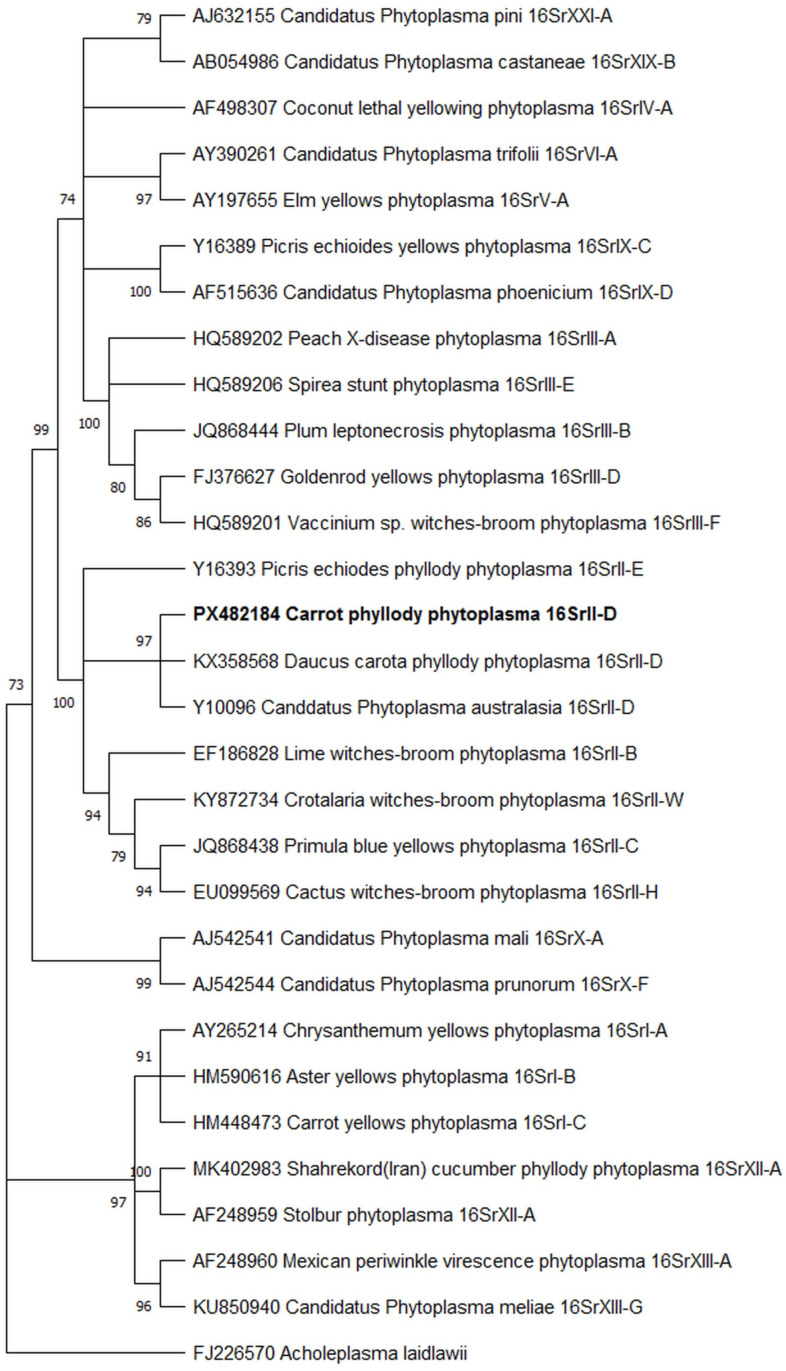
Phylogenetic tree based on 16S rRNA gene sequences. The black carrot phytoplasma isolate obtained in this study (PX482184) clustered within the 16SrII (peanut witches’ broom) group and grouped with reference phytoplasmas assigned to the 16SrII-D subgroup, including Daucus carota phyllody phytoplasma (KX358568) and ‘Candidatus Phytoplasma australasia’ (Y10096). Bootstrap values ≥70% are shown at the nodes; the scale bar indicates evolutionary distance. *Acholeplasma laidlawii* (FJ226570) was used as the outgroup. The sequence generated in this study is indicated in bold.

Phylogenetic analysis based on 16S rRNA gene sequences placed the black carrot phytoplasma isolate (PX482184) within the 16SrII (peanut witches’ broom) group (Figure 2). The isolate clustered with reference phytoplasma sequences assigned to the 16SrII-D subgroup, including Daucus carota phyllody phytoplasma (KX358568) and ‘Candidatus Phytoplasma australasia’ (Y10096). Reference sequences representing other phytoplasma groups, including 16SrI, 16SrIII, 16SrV, 16SrX, and 16SrXII, formed distinct clades separated from the black carrot isolate. The clustering pattern was consistent with the BLASTn results and supported the placement of the detected phytoplasma within the 16SrII-D subgroup (Figure 2).

### 3.5. Phylogenetic Analysis Based on the secA Gene

**Figure 3:**
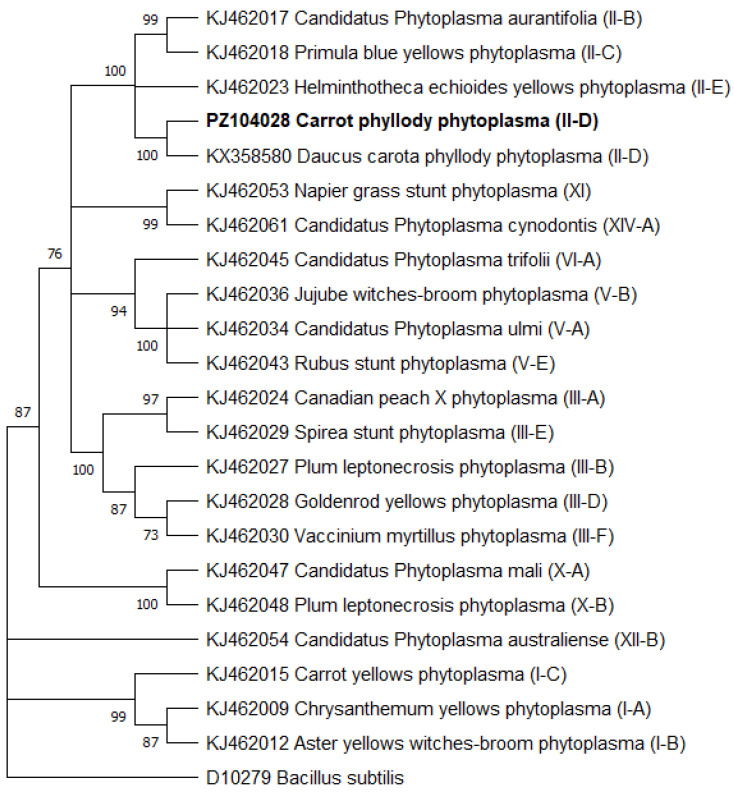
Phylogenetic tree based on *secA* gene sequences. The black carrot phytoplasma isolate obtained in this study (PZ104028) clustered within the 16SrII-D subgroup and grouped closely with Daucus carota phyllody phytoplasma (KX358580). Bootstrap values ≥70% are shown at the nodes, and the scale bar represents evolutionary distance. *Bacillus subtilis* (D10279) was used as the outgroup. The sequence generated in this study is indicated in bold.

Phylogenetic analysis based on the *secA* gene region placed the black carrot phytoplasma isolate (PZ104028) within the 16SrII (peanut witches’ broom) group (Figure 3). The isolate clustered with reference sequences assigned to the 16SrII-D subgroup, including Daucus carota phyllody phytoplasma (KX358580). Other 16SrII subgroup references, such as II-B, II-C, and II-E, were positioned within the broader 16SrII lineage but were separated from the black carrot isolate. The *secA*-based topology was consistent with the 16S rRNA results and provided additional phylogenetic support for the placement of the detected phytoplasma within the 16SrII-D lineage (Figure 3).

### 3.6. tuf Gene-Based Phylogenetic Analysis

**Figure 4:**
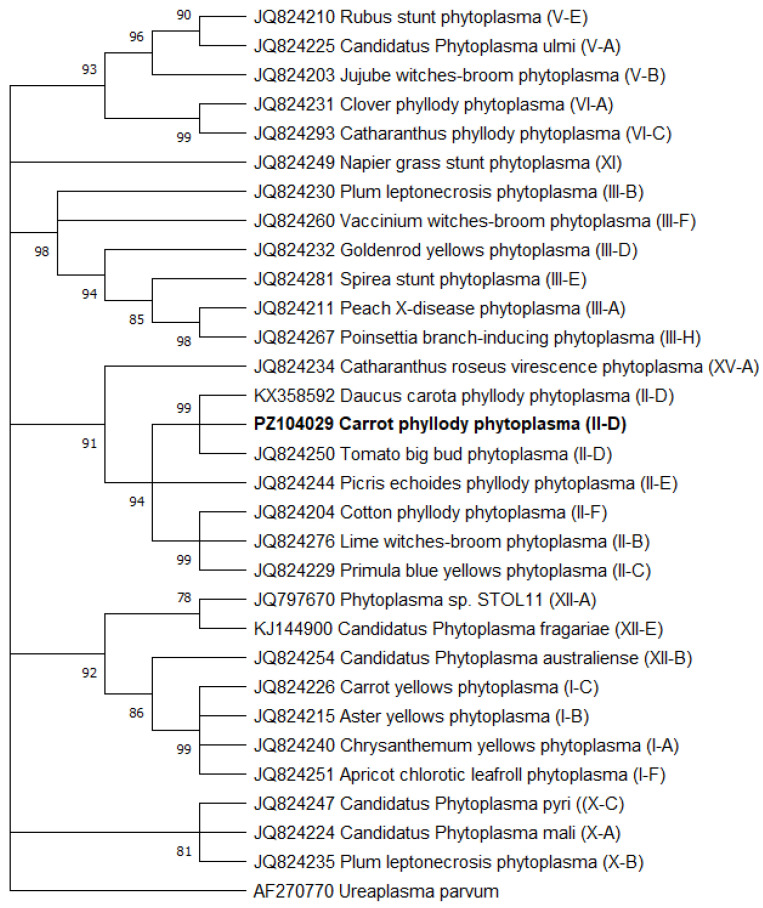
Maximum-likelihood phylogenetic tree based on *tuf* gene sequences. The black carrot phytoplasma isolate obtained in this study (PZ104029) clustered within the 16SrII (peanut witches’ broom) lineage together with reference phytoplasma sequences and grouped closely with Daucus carota phyllody phytoplasma (KX358592) and tomato big bud phytoplasma (JQ824250), both belonging to the 16SrII-D lineage. Bootstrap support values (1000 replicates) ≥70% are shown at the nodes. *Ureaplasma parvum* (AF270770) was used as the outgroup. The sequence generated in this study is indicated in bold.

Phylogenetic analysis based on the *tuf* gene placed the black carrot phytoplasma isolate (PZ104029) within the 16SrII (peanut witches’ broom) lineage together with representative reference phytoplasma sequences (Figure 4). The isolate clustered closely with reference sequences belonging to the 16SrII-D lineage, including Daucus carota phyllody phytoplasma (KX358592) and tomato big bud phytoplasma (JQ824250). Reference sequences representing other phytoplasma groups formed distinct lineages separated from the black carrot isolate. The clustering pattern obtained from the *tuf* gene was generally consistent with those inferred from the 16S rRNA and *secA* analyses and provided additional phylogenetic support for the placement of the detected phytoplasma within the 16SrII-D lineage (Figure 4).

### 3.7. Phylogenetic Analysis Based on the imp Gene

**Figure 5:**
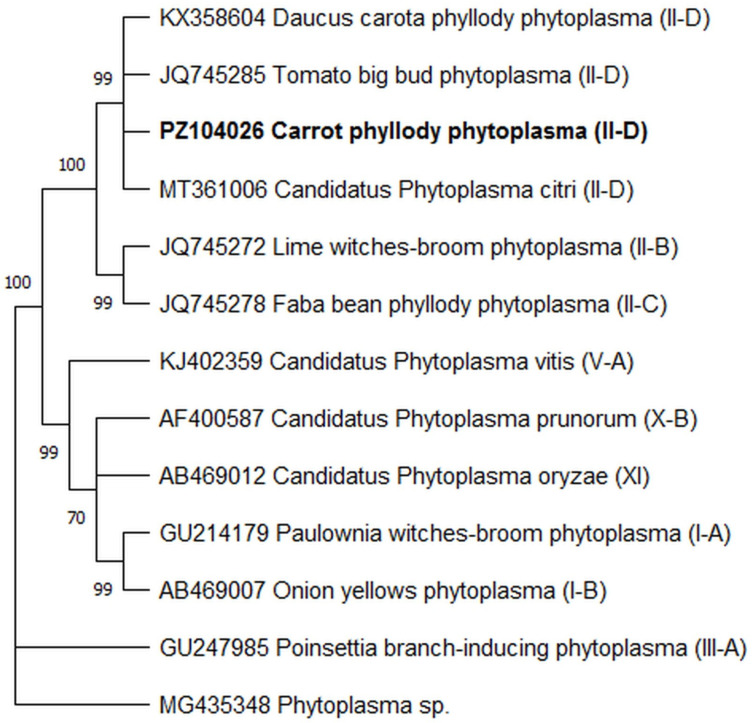
Maximum-likelihood phylogenetic tree based on the *imp* gene region. The black carrot phytoplasma isolate obtained in this study (PZ104026) clustered with reference phytoplasma sequences belonging to the 16SrII-D lineage, including Daucus carota phyllody phytoplasma (KX358604), tomato big bud phytoplasma (JQ745285), and ‘Candidatus Phytoplasma citri’ (MT361006). Bootstrap support values (1000 replicates) ≥70% are shown at the nodes, and the scale bar indicates evolutionary distance. *Phytoplasma* sp. (MG435348) was used as the outgroup. The sequence generated in this study is indicated in bold.

Phylogenetic analysis based on the *imp* gene placed the black carrot phytoplasma isolate (PZ104026) within the 16SrII lineage together with representative phytoplasma reference sequences (Figure 5). The isolate clustered closely with reference sequences belonging to the 16SrII-D lineage, including Daucus carota phyllody phytoplasma (KX358604), tomato big bud phytoplasma (JQ745285), and ‘Candidatus Phytoplasma citri’ (MT361006). Reference sequences representing other phytoplasma lineages formed distinct clusters separated from the black carrot isolate. The clustering pattern obtained from the *imp* gene was generally consistent with those inferred from the 16S rRNA, *secA*, and *tuf* analyses and provided additional phylogenetic support for the placement of the detected phytoplasma within the 16SrII-D lineage (Figure 5).

Taken together, field symptom observations and molecular diagnostic data indicate that the phytoplasma detected in black carrot plants from Hatay belongs to the peanut witches’ broom subgroup (16SrII-D). This assignment is supported by concordant evidence from PCR amplification, sequence analysis, BLASTn comparisons, and phylogenetic analyses based on the 16S rRNA, *secA*, *tuf*, and *imp* gene regions.

## 4. Discussion

This study provides molecular evidence for the presence of a phytoplasma associated with symptomatic black carrot (*Daucus carota* subsp. *sativus*) plants in Hatay province during the 2024 growing season and presents its multilocus molecular characterization. The symptoms recorded under field conditions, including leaf chlorosis and reduced leaf size, shoot proliferation, abnormal fibrous root development with multiple lateral taproots, and phyllody in reproductive organs, are consistent with developmental abnormalities commonly reported in phytoplasma-infected plants [3]. The detection of phytoplasma DNA in all symptomatic samples, together with negative results from asymptomatic controls, supports the association between the observed symptoms and phytoplasma infection. Given the intensive vegetable production in Hatay, these findings indicate the need for further studies on possible inoculum sources, insect vectors, and local disease spread.

Phytoplasmas belonging to the 16SrII (peanut witches’ broom) group have been reported from numerous cultivated and wild plant species and are regarded as among the most widely distributed phytoplasma lineages in several regions of Asia and the Middle East [21,22,29]. Their occurrence in multiple host species increases the likelihood of persistence within agricultural ecosystems and may facilitate pathogen spread through shared insect vectors and alternative host plants [21,22]. The detection of a 16SrII-D phytoplasma in black carrot in Hatay expands the known host range of this subgroup in the region and highlights the need for further studies investigating potential reservoir hosts and vector-mediated transmission pathways under local production conditions.

Because phytoplasmas cannot be cultured in vitro, their detection and classification rely largely on molecular evidence. The 16S rRNA gene remains the primary marker for phytoplasma group and subgroup assignment within the established 16Sr classification framework [8,9]. However, its relatively conserved nature may limit the resolution of relationships among closely related phytoplasma strains. Therefore, additional genetic markers such as *secA*, *tuf*, and *imp* have been widely used to provide improved phylogenetic resolution and finer discrimination among closely related phytoplasma isolates and taxa [10,12].

In the present study, sequence analysis of the 16S rRNA gene provided the primary evidence for assigning the detected phytoplasma to the peanut witches’ broom group, subgroup 16SrII-D. However, the additional *secA*, *tuf*, and *imp* markers increased the reliability of this assignment by providing independent confirmation across loci with different levels of sequence conservation. In particular, the *secA*-based tree appeared to provide the clearest subgroup-level separation, as the black carrot isolate clustered with the 16SrII-D carrot reference isolate and was separated from other 16SrII subgroups, including II-B, II-C, and II-E. This topology provided stronger subgroup-level resolution than the more conserved 16S rRNA region, which is useful for primary classification but may have limited discriminatory power among closely related phytoplasma lineages. The *tuf* and *imp* trees also supported the same placement within the 16SrII-D lineage, confirming that the taxonomic assignment was not dependent on a single marker. Thus, the multilocus approach did not reveal additional within-population diversity in this survey but strengthened the diagnostic confidence and phylogenetic robustness of the 16SrII-D identification.

Phytoplasma infections of carrot have been reported from various regions worldwide and have been associated with several phytoplasma groups, including 16SrI, 16SrII, and 16SrXII [13,16,17,18,30]. Among these, aster yellows-related phytoplasmas (16SrI) and stolbur phytoplasmas (16SrXII) are among the most frequently reported agents in carrot [13,30]. In contrast, reports of carrot-associated phytoplasmas belonging to the peanut witches’ broom group (16SrII) remain relatively limited and are largely restricted to countries in the Middle East and Asia [16,17,18]. Previous studies have documented 16SrII phytoplasmas associated with witches’ broom, phyllody, yellowing, and related symptoms in carrot crops in Iran, India, and Indonesia [16,17,18]. In the present study, the phytoplasma detected in symptomatic black carrot plants from Hatay was assigned to subgroup 16SrII-D based on 16S rRNA sequence analysis and supported by multilocus phylogenetic evidence. Together with previous reports, these findings indicate that carrot can serve as a host for diverse members of the 16SrII lineage and further expand the current knowledge of carrot-associated phytoplasmas in the region.

The detection of 16SrII-D phytoplasma in black carrot may have practical implications for regional vegetable production. If black carrot supports pathogen multiplication and is exposed to competent leafhopper vectors, it could potentially act as an additional reservoir host within mixed vegetable production systems. This is particularly relevant for Hatay, where intensive and diverse vegetable cultivation may facilitate interactions among cultivated hosts, weeds, and insect vectors. Therefore, future studies should test whether black carrot can contribute to the infection cycle by combining systematic field surveys with molecular screening of symptomatic and asymptomatic black carrot plants, neighboring vegetable crops, weed species, and phloem-feeding insects. Vector transmission assays using candidate leafhopper species, graft- or dodder-mediated transmission experiments, and seasonal monitoring of phytoplasma prevalence in plants and insects would help clarify the host range, inoculum sources, and spread dynamics of the detected 16SrII-D phytoplasma.

Phytoplasmas of the 16SrII group are transmitted by phloem-feeding insect vectors, particularly leafhoppers, which play a key role in the epidemiology of witches’ broom-type diseases. In carrot production systems, *Orosius albicinctus* has been reported as an important vector associated with carrot witches’ broom and related phytoplasma diseases [16]. Previous studies have also shown that this vector can acquire and transmit phytoplasmas among different host plants, contributing to pathogen dispersal within agricultural landscapes [16]. In addition, 16SrII-D phytoplasmas have been reported from multiple vegetable crops in the region, indicating a relatively broad host range and suggesting the existence of alternative plant reservoirs [22]. Therefore, the occurrence of a 16SrII-D phytoplasma in black carrot fields in Hatay highlights the importance of future studies focusing on potential insect vectors, alternative host plants, and possible inoculum sources involved in local disease epidemiology. Such information would be valuable for understanding phytoplasma persistence and spread within regional production systems. The value of integrating molecular characterization with phylogenetic analyses for improving phytoplasma identification and resolving relationships among closely related taxa has also been demonstrated previously [11,12]. From a disease management perspective, integrated phytoplasma management should include regular field monitoring, removal of symptomatic plants to reduce potential inoculum sources, management of weed hosts that may serve as reservoirs, and control of phloem-feeding insect vectors, particularly leafhoppers. In addition, the use of pathogen-free planting materials and avoidance of planting near heavily infested or unmanaged host vegetation may help reduce the risk of local disease spread.

SAP11 is among the best-characterized phytoplasma effector proteins and has been associated with symptom development in several phytoplasma pathosystems through its interactions with host regulatory pathways [19,20]. In the present study, SAP11 was included as an additional molecular marker and was successfully detected in symptomatic black carrot samples. However, no functional analyses or comparative sequence investigations were performed. Therefore, the potential contribution of SAP11 to symptom development in black carrot cannot be determined from the current data and remains a subject for future research.

The absence of sequence polymorphism among the 23 symptomatic samples suggests that the analysed phytoplasma population was homogeneous across the targeted loci within the surveyed fields. However, this result should be interpreted cautiously because only selected marker regions were analysed, and whole-genome-level diversity was not assessed. In addition, the limited number of asymptomatic plants included as negative controls does not allow conclusions regarding latent infections in apparently healthy black carrot plants. Future studies should include larger-scale sampling of both symptomatic and asymptomatic plants, vector populations, and alternative host plants to evaluate genetic diversity, latent infection frequency, and local epidemiological patterns.

While the multilocus molecular approach employed in this study provided consistent molecular evidence supporting the identification of the detected phytoplasma as a member of the 16SrII-D subgroup, pathogenicity assays and experimental transmission studies were not conducted. Because phytoplasmas are unculturable pathogens and their biological characterization generally requires insect-vector transmission or graft-inoculation experiments, such investigations were beyond the scope of the present study. In addition, a positive control was not available during PCR analyses. Nevertheless, all symptomatic samples consistently yielded the expected amplicons for the analysed loci, whereas asymptomatic plants and no-template controls remained negative, and sequence analyses further confirmed the phytoplasma identity. Therefore, although a strong association was observed between the detected phytoplasma and the symptoms recorded in black carrot plants, a direct causal relationship was not experimentally demonstrated. Future studies should therefore include controlled transmission assays with candidate leafhopper vectors, graft- or dodder-mediated inoculation experiments to verify pathogenicity in black carrot, molecular screening of weeds and neighboring commercial crops to identify alternative reservoir hosts, and multi-season surveys to determine disease prevalence and seasonal dynamics. Such experiments would help clarify the infection cycle, host plant range, vector involvement, and potential routes of pathogen spread in regional production systems.

Overall, this study provides molecular and phylogenetic evidence for the occurrence of a 16SrII-D phytoplasma associated with symptomatic black carrot plants in Hatay, Türkiye. This finding expands the current knowledge of carrot-associated 16SrII phytoplasmas and suggests that black carrot may represent an additional host within regional production systems. Given the relatively limited number of reports of 16SrII phytoplasmas in carrot [16,17,18] and the detection of 16SrII-D phytoplasmas in several vegetable crops in the region [22], the present finding is relevant for local plant health monitoring. Future studies should prioritize identifying potential insect vectors, screening alternative host plants and weeds as possible inoculum reservoirs, assessing disease distribution across production areas, and conducting multi-season surveys to estimate prevalence and seasonal dynamics. Such studies will be important for understanding the epidemiology of this phytoplasma and for developing appropriate monitoring strategies in black carrot production systems.

## 5. Conclusions

This study provides the first documented evidence of a 16SrII-D phytoplasma associated with black carrot in Türkiye and documents black carrot as a newly recognized host associated with this phytoplasma subgroup in the country. The main scientific message of this work is that black carrot production areas should be included in phytoplasma surveillance and monitoring programs, particularly in regions where mixed vegetable cultivation, weed hosts, and phloem-feeding insect vectors may facilitate pathogen persistence and spread. Because black carrot is economically important for both fresh-market consumption and anthocyanin-based industrial processing, phytoplasma-associated abnormalities in root development and plant morphology may negatively affect crop quality, marketability, and sustainable production.

The detection of 16SrII-D phytoplasma in black carrot further suggests that this crop may serve as a potential inoculum reservoir for other commercial vegetable crops grown in the same production systems. Therefore, this finding has important diagnostic, epidemiological, and plant protection implications beyond a single host record. The multilocus molecular approach combining PCR, sequencing, BLASTn comparison, and phylogenetic analysis provides a robust diagnostic framework for reliable subgroup-level identification, although broader population-level studies are still required. Future research should focus on determining the epidemiological role of black carrot as a potential reservoir host, identifying competent insect vectors, screening neighboring vegetable crops and weed species, and assessing disease prevalence through multi-season surveys. Whole-genome sequencing and multilocus sequence analysis (MLSA) should also be employed to investigate population diversity, strain relationships, and epidemiological links among 16SrII-D phytoplasma isolates from different hosts and geographic regions.

## Figures and Tables

**Table 1:** Primers used for PCR amplification of phytoplasma genes.

Target Gene	Primer Name	Primer Sequence (5′-3′)	Expected Amplicon Size (bp)	Reference
16S rRNA	P1/P7	AAGAGTTTGATCCTGGCTCAG/ CGTCCTTCATCGGCTCTT	1800	[24]
16S rRNA	R16F2n/R16R2	GAAACGACTGCTAAGACTGG/ TGACGGGCGGTGTGTACAAACCCCG	1250	[25]
*secA*	SecAfor1/SecArev3	ATGAAAAACGTGAGTGGTTT/ CCTTCATTTGACGAGGTTTT	480	[11]
*tuf*	fTuf1/rTuf1	CCTGAAGAAAGAGAAGACTA/ CGGAAATAGAATTAGTTGGT	940	[26]
*imp*	ImpF/ImpR	GAGCTTTGGTGGTGTTTTAG/ CCAATGTTGCCTTTGATACC	520	[27]
SAP11	SAP11-F/SAP11-R	ATGTTAGTTGTTGATGAGG/ TTAGGCGTTGTTTCTTTGC	330	[28]

## Data Availability

The nucleotide sequence datasets generated in this study are available in the NCBI GenBank repository under accession numbers PX482184 (16S rRNA), PZ104026 (*imp*), PZ104028 (*secA*), PZ104029 (*tuf*), and PZ104027 (SAP11). Other datasets generated or analysed during the current study are available from the corresponding author upon reasonable request.
